# 全身坏死性淋巴结炎1例

**DOI:** 10.3760/cma.j.cn121090-20250702-00309

**Published:** 2025-10

**Authors:** 林玲 付, 珊 孙, 丹丹 王, 佳楠 李

**Affiliations:** 解放军总医院第六医学中心耳鼻咽喉头颈外科医学部，北京 100048 Department of Otorhinolaryngology, Head and Neck Surgery, the Sixth Medical Center of the Chinese PLA General Hospital, Beijing 100048, China

患者男，24岁，因“发热6 d，双耳垂周围肿胀3 d”入院。患者无明显诱因发热，体温>38.5 °C，多于午后和夜间发热，伴乏力、食欲下降、咽痛，轻微咳嗽、双侧颈部疼痛，于2025年1月9日起病。发病3 d后开始出现双耳垂周围肿胀及疼痛，无张口受限等不适。曾就诊于外院，诊断为急性腮腺炎，给予布洛芬药物对症处理。

查体：双耳垂周围轻度弥漫性隆起、皮肤轻微充血、皮温升高、局部压痛。咽部轻微充血。双侧颈部可触及多处小淋巴结肿大，轻压痛。实验室检查：传染病四项筛查阴性；生化指标正常；血、尿淀粉酶正常。呼吸道8项病原IgM均阴性。风湿免疫相关指标中，补体C4升高，其余均正常。甲功、肿瘤标志物、结核抗体检查均正常。血培养结果阴性。EB病毒抗体检测：EB病毒核抗原及衣壳抗原IgG阳性，早期抗原IgG可疑阳性，衣壳抗原IgM抗体阴性。单纯疱疹病毒检测阴性。而血常规WBC及感染指标随病情进展变化：发病第6天白细胞正常，第12天各类白细胞均降低（WBC 2.45×10^9^/L、ANC 1.41×10^9^/L、淋巴细胞计数0.86×10^9^/L、单核细胞计数0.17×10^9^/L、嗜酸性粒细胞计数0、嗜碱性粒细胞计数0.01×10^9^/L），第15天WBC有所恢复，但单核细胞较前有所下降。发病第8、13、15天检查感染指标：降钙素原正常；IL-6及CRP轻度升高，且随着病情进展，数值持续上升，发病第15天，IL-6：9.5 pg/ml（参考范围：0～6.4 pg/ml），CRP：19.4 mg/L（参考范围：0～5 mg/L）。随着病情进展，中性粒细胞CD64感染指数升高并超出正常范围，而CD14单核细胞百分比则持续下降。影像学检查：腮腺超声检查提示双侧腮腺局部回声欠均匀，但双侧颌下及腮腺周边可见多个低回声结节，考虑增大淋巴结。肺CT未见异常。颅脑核磁平扫未见异常。PET-CT见双侧腮腺深浅叶、双侧颈部及锁骨上窝、左侧上臂皮下及肌间隙内、双侧腋下、右侧肺门区、肝门、门腔间隙、腹膜后、肠系膜上、双侧髂血管周围、盆底及双侧腹股沟见多发增大淋巴结，部分周围脂肪间隙模糊，较大短径约1.4 cm，^18^F-氟代脱氧葡萄糖（FDG）摄取均增高，较高SUV平均值/最大值约14.6/23.0。结论：全身多发多组增大淋巴结，葡萄糖代谢增高，考虑淋巴组织增生性病变，与感染相关（如病毒等）或非特异性淋巴结炎可能性大，应取右侧腋窝淋巴结穿刺活检排除淋巴瘤。双侧腋窝超声检查可见多个低回声结节，右侧较大结节4.0 cm×1.9 cm，左侧较大结节2.3 cm×1.2 cm。右侧腋窝淋巴结超声引导下粗针穿刺活检，活检病理提示：不规则坏死伴组织细胞及吞噬核碎屑，可见浆细胞样单核细胞，周围以T淋巴细胞为主的淋巴细胞增生，免疫组化CD123阳性（浆细胞样单核细胞），CD68阳性（组织细胞和浆细胞样单核细胞）；分子原位杂交EB病毒编码RNA阴性，考虑坏死性淋巴结炎。

患者入院后给予注射用头孢呋辛钠（1.5 g，2次/d）、利巴韦林、连花清瘟颗粒、蓝芩口服液、吸入用布地奈德混悬液雾化治疗，布洛芬缓释胶囊对症降温处理。发病第11天，患者双耳垂周围肿胀及疼痛、咽痛缓解，但仍间断性发热，体温>39 °C。停用头孢呋辛钠、利巴韦林、连花清瘟颗粒及激素雾化药物，继续口服蓝芩口服液、板蓝根颗粒。发病第15天，患者食欲明显下降，停用所有口服药物。发病第18天患者出院，出院时仅有发热症状，体温最高约38 °C，较前症状减轻。患者出院后未继续服用相关药物，发病第25天其发热、头痛症状完全缓解。发病第35天复查血常规、生化指标均正常。发病第55天复查浅表部位淋巴结超声：腹股沟淋巴结、颈部淋巴结均未见异常肿大淋巴结，腋窝淋巴结肿大但较前缩小。发病5个月后电话随访，患者一般状态良好。

